# Multi-Chamber Reverse Remodeling and Hemodynamic Force Realignment After SGLT2 Inhibitor Initiation in Real-World Heart Failure

**DOI:** 10.3390/jcdd13060260

**Published:** 2026-06-11

**Authors:** Silvia Prosperi, Sara Monosilio, Andrea D’Amato, Danilo Angotti, Domenico Filomena, Lucrezia Netti, Giovanni Tonti, Gianni Pedrizzetti, Sara Cimino, Roberto Badagliacca, Paolo Severino, Carmine Dario Vizza, Viviana Maestrini

**Affiliations:** 1 Department of Clinical, Internal, Anesthesiologic and Cardiovascular Sciences, La Sapienza University of Rome, 00161 Rome, Italy; silvia.prosperi@uniroma1.it (S.P.); sara.monosilio@uniroma1.it (S.M.); andrea.damato@uniroma1.it (A.D.); danilo.angotti@uniroma1.it (D.A.); domenico.filomena@uniroma1.it (D.F.); lucrezia.netti@uniroma1.it (L.N.); sara.cimino@uniroma1.it (S.C.); roberto.badagliacca@uniroma1.it (R.B.); paolo.severino@uniroma1.it (P.S.); dario.vizza@uniroma1.it (C.D.V.); 2 Cardiology Division, G. D’Annunzio University, 66100 Chieti, Italy; giatonti@gmail.com; 3 Department of Engineering and Architecture, University of Trieste, 34127 Trieste, Italy; gianni.pedrizzetti@dia.units.it

**Keywords:** hemodynamic forces, strain imaging, heart failure, cardiac reverse remodeling, SGLT2i

## Abstract

Background: Sodium–glucose cotransporter 2 inhibitors (SGLT2i) promote beneficial effects on cardiac reverse remodeling (RR) in heart failure (HF). However, most imaging evidence focuses on single chambers, mainly the left ventricle (LV) or left atrium (LA), whereas integrated biventricular and atrial remodeling remains less explored. Moreover, real-world data are limited, and myocardial–flow coupling markers, such as hemodynamic forces (HDFs), are scarcely investigated, with uncertain sex-related differences. Purpose: To evaluate multi-chamber cardiac RR after SGLT2i initiation in a real-world HF population. Secondary aims are to assess whether changes in HDFs provide additional functional insight into myocardial–flow coupling beyond conventional echocardiographic indices, and to descriptively explore sex-related differences in echocardiographic remodeling. Methods: Patients with HF and ejection fraction (EF) ≤ 45%, naive to SGLT2i and on stable guideline-directed medical therapy for ≥3 months, were enrolled. Standard and advanced echocardiography were performed at baseline and follow-up, including speckle-tracking and HDFs assessment. NYHA class and NT-proBNP were collected. Analyses were performed overall and stratified by sex. Results: Sixty-eight patients were included. After 6 months, RR was observed across all chambers: LV-RR in 33 patients (49%), right ventricular (RV) RR in 35 (52%), biventricular RR in 18 (27%), and LA-RR in 14 (21%). HDFs showed significant realignment, suggesting association with improved myocardial–flow coupling. RR effects were comparable between sexes (*p* > 0.05). NT-proBNP significantly decreased. Conclusions: In this real-world cohort, SGLT2i therapy was associated with significant multi-chamber RR and HDFs realignment, supporting improved myocardial–flow coupling beyond conventional indices. Exploratory sex-related analyses showed no significant differences. Larger and longer-term randomized studies are warranted.

## 1. Introduction

Cardiac remodeling is a pathological process characterized by structural and functional alterations in response to chronic stressors such as volume or pressure overload or myocardial infarction, ultimately contributing to the progression of heart failure (HF). This process involves changes in cardiac size, shape, and function, which are key determinants of HF progression and are associated with adverse clinical outcomes and poor prognosis [1]. Inhibiting, or at least attenuating, these pathological processes are fundamental to the beneficial effects of many HF therapies.

Sodium-Glucose Cotransporter-2 Inhibitors (SGLT2i) are a class of pharmacological agents initially developed for the treatment of type 2 diabetes mellitus. Beyond their glucose-lowering effects, SGLT2i have demonstrated cardioprotective and nephroprotective properties, reducing HF-related hospitalizations, cardiovascular mortality, and the progression of chronic kidney disease. These benefits have positioned SGLT2i as a cornerstone therapy in HF management, regardless of the presence of diabetes.

SGLT2i are pleiotropic agents with a broad spectrum of effects beyond glycemic control, and among HF therapies they exhibit particularly “eclectic” mechanisms of action. These include metabolic modulation as a primary effect, together with anti-fibrotic, anti-inflammatory, and anti-oxidative stress properties, as well as reductions in preload and afterload through osmotic diuresis and natriuresis [2]. Through this multi-target profile, they may be expected to favorably influence the function and structure of all cardiac chambers, contributing to global reverse remodeling.

Most available studies on SGLT2i have predominantly focused on clinical endpoints such as mortality and hospitalization, showing significant benefits [3]. The impact on cardiac reverse remodeling has been investigated in multiple studies, with some limitations. Analyses have primarily focused on isolated echocardiographic parameters or on the remodeling of individual cardiac chambers, rather than providing a comprehensive assessment of global cardiac structural and functional changes. Furthermore, imaging studies consider mainly ejection fraction (EF) and/or strain as indices of systolic function, while modern parameters for assessing cardiac function—such as hemodynamic forces (HDFs) for the evaluation of the myocardium–flow coupling—have not been considered. Considering the different pathways through which favorable cardiac remodeling can express itself may aid in elucidating the complexity of the mechanisms involved and in better quantifying the actual therapeutic impact.

In addition, it has to be considered that sex-specific differences in response to therapy are plausible due to the possible influence by hormones, body composition, and distinct patterns of cardiac remodeling in males and females [4]. However, only few studies have investigated sex-based influences on cardiac remodeling and without focusing on SGLT2i.

The primary objective of this study is to assess the effect of SGLT2i on cardiac reverse remodeling (RR) evaluated by advanced echocardiography after 6 months of therapy. The secondary aim is to explore whether changes in HDFs provide complementary functional information beyond conventional remodeling parameters and to descriptively assess sex-related differences in cardiac RR.

## 2. Materials and Methods

### 2.1. Study Design and Protocol

This is a single-center, prospective, observational study conducted at Policlinico Umberto 1, University Hospital of Sapienza University of Rome. All consecutive patients diagnosed with chronic HF with LV-EF ≤ 45% who started therapy with SGLT2i (Empagliflozin or Dapagliflozin) according to current guidelines [5,6], from November 2022 to November 2024, were screened. Prior to starting SGLT2i, patients had already been receiving optimal HF medical for at least 3 months, according to their tolerance and to ESC Guidelines [5,6]. The inclusion of patients with baseline LV-EF ≤ 45% was chosen to focus on a cohort of patients with reduced or mildly reduced systolic function, where reverse remodeling is more likely to occur and can be accurately quantified over follow-up. Exclusion criteria included previous administration or contraindication to SGLT2i, known allergies to SGLT2 inhibitors, pregnancy or breastfeeding, and discontinuation of treatment. The present analysis included only patients with complete echocardiographic assessment at T0 and T1. Patients without available follow-up were excluded from paired analysis.

Before starting SGLT2i therapy (T0) and after 6 months of therapy (T1), all recruited patients underwent clinical assessment with New York Heart Association (NYHA) Class estimation, blood tests including N-terminal pro B-type Natriuretic Peptide (NT-proBNP) and echocardiographic evaluation. Concomitant HF therapies, including ACE inhibitors/ARNI, beta-blockers, mineralocorticoid receptor antagonists, and diuretics, were recorded at baseline and during follow-up.

The echocardiographic evaluation included the quantification of: left ventricular EF (LV-EF), left ventricular end-diastolic volume (LV-EDV), left ventricular end-systolic volume (LV-ESV), left ventricular longitudinal and circumferential strain (LV-GLS and LV-GCS), diastolic function (E/e’), global longitudinal strain of the right ventricle (RV-GLS), strain of right free-wall (RFW-St), right ventricular fractional area change (RV-FAC), left atrial ejection fraction (LA-EF), left atrial longitudinal strain (LA-GLS) and left atrial end systolic volumes (LA-ESV). All these parameters were calculated according to current guidelines using specialized software (QStrain, Medis BV, version 1.4, Leiden, The Netherlands). The HDFs, which represent the energy exchanged between the blood and the endocardial surface, were computed using dedicated software (QStrain, Medis BV, Leiden, The Netherlands), based on a previously validated mathematical model and already described [7,8,9,10]. LV endocardial borders were semi-automatically traced in all three long-axis views, both during systole and diastole, with manual correction when required to ensure adequate tracking. The model utilizes LV endocardial displacement, along with the estimated mitral and aortic valve areas, as input data for the calculation of HDFs, generating global intraventricular force vectors averaged over the LV blood volume. HDFs were evaluated throughout the entire cardiac cycle and separately during systole and diastole, in both longitudinal (apex-to-base; A–B) and transverse (latero-septal; L–S) directions. The predominant orientation of intraventricular forces was determined by calculating the ratio of L–S to A–B HDFs (L–S/A–B HDFs ratio, %). The L–S/A–B ratio was computed for systole, diastole, and the whole cardiac cycle, and used as the main index of force orientation. Cases with inadequate image quality or unreliable tracking were excluded from HDF analysis. All strain and HDFs analyses were performed offline by blind experienced operators following a standardized protocol, with manual border correction when required to ensure adequate tracking quality.

The primary aim of this study is to evaluate the effect of SGLT2i after 6 months on cardiac remodeling using advanced echocardiographic parameters.

The primary outcome is to assess the extent of reverse remodeling by comparing the echocardiographic parameters between baseline (before starting SGLT2i therapy, T0) and the first follow-up (after 6 months of therapy, T1). 

Composite reverse remodeling endpoints were intentionally defined using a stringent definition to capture meaningful structural and functional improvement and to minimize misclassification due to measurement variability or regression to the mean. The composite endpoints to define a significant reverse remodeling involving LV, RV and LA are as follows:

- LV reverse remodeling (LVRR) was defined as the simultaneous presence of all the following conditions, according to previous studies [9,11]: an increase in LV-EF ≥ 10%, a reduction in LV-ESV ≥ 15%, and an absolute increase in LV-GLS ≥ 5%.
- RV reverse remodeling (RVRR), in the absence of an established definition, was defined as the simultaneous presence of all the following conditions: an increase in absolute RV-GLS ≥ 5%, an increase in absolute RFW-St ≥ 5%, and an increase in RV-FAC ≥ 5%.
- LA reverse remodeling (LARR), in the absence of an established definition, was defined as the simultaneous presence of all the following conditions: an increase in LA-EF ≥ 10%, a reduction in LA-ESV ≥ 15%, and an absolute increase in LA-GLS ≥ 5%.

Cut-offs were defined a priori and selected to exceed expected intra-observer variability. The chosen thresholds were selected to exceed the expected measurement variability reported in prior reproducibility studies [12,13,14] as shown in Supplementary Materials Table S1, and in order to increase the likelihood that the observed changes reflected clinically meaningful remodeling rather than technical variability alone. Nevertheless, these thresholds remain empirical and require validation in larger dedicated studies.

The secondary aims were to assess whether changes in HDFs provide complementary functional information beyond conventional remodeling parameters and to descriptively assess eventual sex-related differences in cardiac reverse remodeling after SGLT2i therapy.

To address the first one, we compared left ventricular HDFs parameters before and after treatment.

Sex-related effects on response to SGLT2i were evaluated in an exploratory manner by comparing the relative change (Δ) in median values of each echocardiographic parameter between male and female patients. No formal sample size or power calculation was performed for between-sex comparisons. Δ was calculated as (T1 − T0)/T0 for each variable. Positive Δ values indicate an increase in the parameter over time. 

One-year clinical outcomes, including arrhythmias, cardiologic admissions, all-cause hospitalizations (ACH), all-cause mortality (ACM), and a composite endpoint, were prospectively collected during follow-up and predefined for analysis.

Intra- and inter-observer reproducibility was assessed in a randomly selected subset of 15 subjects by repeated offline analyses performed by the same observer and by a second independent observer, respectively. Reproducibility was evaluated using intraclass correlation coefficients (ICCs) and corresponding 95% confidence intervals (CIs).

### 2.2. Statistical Analysis

Data were statistically analyzed using the Statistical Package for Social Sciences, version 27.0 (SPSS, Chicago, IL, USA). The variables have been analyzed to test a normal distribution using the QQ plot method. Continuous variables are presented as mean and SD or median (25th–75th percentiles) and were compared using Student’s *t*-test or the Mann–Whitney rank sum as appropriate. The categorical variables are expressed as counts and percentages and were compared using the chi-square test. Paired comparisons of continuous variables were performed with two-tailed paired Student’s *t*-test or Wilcoxon test, when appropriate. Paired comparisons of categorical variables were conducted with the McNemar test. Logistic regression analysis (univariate and multivariate) was used to explore variables associated with biventricular and atrial reverse remodeling. All variables with a *p* value of 0.10 or less at univariate analysis were considered for the multivariate models. Differences were considered statistically significant when the *p* value was less than 0.05. Given the exploratory nature of the study and the relatively limited sample size, no formal correction for multiple comparisons was applied, in order to avoid excessive type II error. Therefore, the results should be interpreted as hypothesis-generating and considered with appropriate caution. Intra and inter-observer reproducibility were evaluated using ICCs with corresponding CIs.

## 3. Results

### 3.1. Baseline Features of the Population

A total of 85 patients were screened, 68 patients (80%) completed the 6-month follow-up and were included in the final analysis. A total of 17 patients were excluded due to inadequate image quality and/or suboptimal endocardial tracking that precluded reliable analysis.

All patients had chronic HF with low EF (≤45%) with ischemic etiology in half of the cases (50%), a mean age of 70 ± 13 years, and 72% were male. Hypertension was present in 48 patients (71%), dyslipidemia in 41 patients (60%), and diabetes in 22 patients (32%). A history of smoking was reported in 35 patients (51%).

In the overall population, the baseline HF medications were predominantly constituted by Beta-Blockers (BB) (87%), Angiotensin Receptor-Neprilysin Inhibitor (ARNi) (68%), Mineralocorticoid Receptor Antagonist (MRA) (68%), and loop diuretics (62%), since the population was mostly composed of HFrEF patients. Renin–Angiotensin–Aldosterone System (RAAS) inhibition was widely implemented, with 89% of patients receiving either ARNi or Angiotensin II Receptor Blocker (ARB) or Angiotensin-Converting Enzyme inhibitors (ACEi). All medical therapies were up titrated to the maximum tolerated doses in accordance with guideline recommendations [5].

At baseline, the most prevalent NYHA Class was II, comprising 45 patients (66%), followed by Class III with 17 patients (25%). The median NT-proBNP value was 1651 (663–1943) pg/mL.

Baseline features are depicted in Table 1.

### 3.2. Echocardiographic Analysis

At baseline, patients exhibited impaired global cardiac function as reflected by elevated volumes and reduced strain and EF values.

At follow-up, significant improvements were observed across most parameters.

The comparison of echocardiographic parameters at baseline (T0) and after 6 months of therapy (T1) is shown in Table 2.

Specifically, both median LV-EDV and median LV-ESV significantly decreased (*p* < 0.001 for both). LV-EF improved significantly (*p* < 0.001), along with LV-GLS and LV-GCS with *p* < 0.001 for both. Also, the diastolic function optimized with the E/e’ significantly decreased at T1 (*p* < 0.001).

RV parameters similarly improved: RV-GLS, RV-FAC (*p* < 0.001) and RFW-St (*p* = 0.001). TAPSE also improved from 19 mm to 20 mm even though it was not statistically significant (*p* = 0.304).

LA function also showed recovery, with a rising of LA-EF, LA-GCS (*p* < 0.001) and LA-GLS (*p* > 0.004). LA-ESV did not significantly change even though it showed a trend in decreasing starting from 76 mL (IQR: 57–97) to 74 mL (IQR: 59–97) (*p* = 0.331).

Reverse remodeling, based on previous definitions, was documented in a considerable portion of patients: LVRR occurred in 33 patients (49%), RVRR in 35 patients (52%), LARR in 14 patients (21%), and biventricular reverse remodeling in 18 patients (27%).

Strain and HDFs parameters in the entire cardiac cycle showed excellent intra- and inter-observer reproducibility across all measurements. Supplementary Materials Table S2, summarizes the intra-operator and inter-operator variability.

In the univariate logistic regression analysis for biventricular reverse remodeling, we included only parameters that were not directly involved in the definition of the outcome itself, to avoid circular reasoning or collinearity. None of the clinical or imaging variables investigated were significantly associated with the study endpoint. Logistic regression for biventricular remodeling is shown in Table 3.

In the univariate analysis for LA reverse remodeling, LV-EDV (OR: 1.01, 95% CI: 1.00–1.02, *p* = 0.043) and LV-ESV (OR: 1.01, 95% CI: 1.00–1.02, *p* = 0.036) were the only variables significantly associated. However, in multivariate analysis, both LV-EDV (OR 1, 95% CI 0.961–1.03, *p* = 0.902) and LV-ESV (OR 1.01, 95% CI 0.97–1.06, *p* = 0.564) lost their statistical significance.

Logistic regression for LA remodeling is shown in Table 4.

### 3.3. Hemodynamic Forces Analysis

HDFs ratios indicated improved myocardium–flow coupling efficiency at follow-up, with the entire cycle ratio decreasing from 31% (IQR: 25–37) to 28% (IQR: 22–33) (*p* = 0.02), systolic ratio from 21% (IQR: 16–26) to 17% (IQR: 12–21) (*p* = 0.001), and diastolic ratio from 47% (IQR: 38–51) to 38% (IQR: 26–44) (*p* < 0.001).

**Figure 1:**
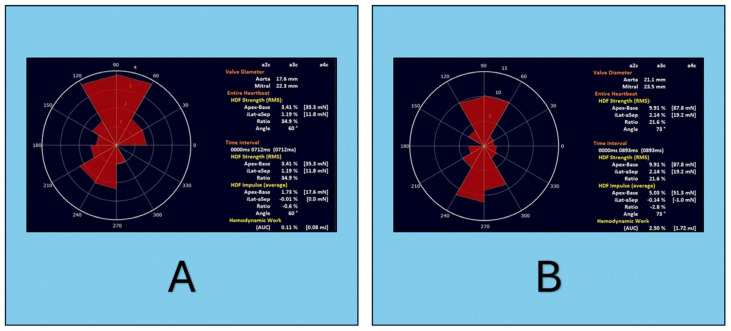
The distribution of haemodynamic forces (HDFs) before SGT2i therapy (**A**) and after 6 months (**B**).

The comparison of HDFs ratios at baseline (T0) and after 6 months of therapy (T1) is shown in Table 2. Figure 1 expresses the redistribution of HDFs after SGLT2i therapy.

At baseline, HDF strength (RMS) showed a predominant component along the apex-to-base axis (A–B: 9.41%, 35.3 mN) with a smaller contribution along the lateral–septal axis (L–S: 1.19%, 11.4 mN), resulting in a ratio of 34.2% and a mean vector angle of 60°, indicating a partially dispersed force alignment. After 6 months of SGLT2i treatment, a more physiological reorientation was documented, characterized by a clear realignment of force vectors toward the A–B direction and an increased predominance of longitudinal forces: A–B RMS remained high (9.91%, 87.8 mN), while the L–S component increased modestly (2.14%, 19.2 mN), with a lower ratio of 21.6% and a larger mean angle of 73°, consistent with a net shift in intraventricular momentum toward the apex-to-base direction. Notably, hemodynamic work (AUC) increased from 0.11% (0.08 mJ) at baseline to 2.90% (1.72 mJ) at follow-up, supporting the presence of a more energetically relevant but better organized flow pattern. Overall, these findings suggest improved hemodynamic efficiency and a more coordinated cardiac mechanical–fluid coupling after therapy.

### 3.4. Sex Difference

After dividing the population into two groups based on sex, we compared the relative median differences (delta, Δ) for most significant echocardiographic parameters between male and female patients, confirming a comparable effect of cardiac reverse remodeling in both groups (*p* > 0.05 for all). The percentages of remodeling were comparable between sexes: biventricular RR was observed in 14 males (29%) and 4 females (21%) (*p* = 0.528); LVRR was observed in 23 males (47%) and 10 females (53%) (*p* = 0.673); RVRR in 27 males (55%) and 8 females (42%) (*p* = 0.336); and LARR in 9 males (18%) and 5 females (26%) (*p* = 0.467). The comparison of echocardiographic relative changes from T0 to T1 is shown in Table 5.

### 3.5. NYHA Class and NT-proBNP Changes

NT-proBNP levels showed a significant decline, with the median value decreasing from 1651 pg/mL (663–1943) at T0 to 946 pg/mL (361–1452) at T1 (*p* = 0.005). The percentage of patients in NYHA class > 2 decreased from 27% at T0 to 18% at T1, although without reaching significance (*p* = 0.21).

In our cohort, the 1-year follow-up showed arrhythmias in 12%, cardiologic admissions in 14%, ACH in 26%, ACM in 6%, and a composite endpoint in 32% of patients.

## 4. Discussion

In this study, we assessed the extent of echocardiographic reverse remodeling in a real-world cohort of 68 patients affected by chronic HF with EF ≤ 45%, all of whom had been on optimized and up-titrated therapy for at least 3 months—except for SGLT2 inhibitors, which were newly initiated—over a 6-month follow-up period after starting this latter treatment.

The main finding of this imaging study, based on an in-depth assessment including both traditional and advanced echocardiography, is the evidence of an association with significant reverse remodeling across multiple cardiac chambers: combined biventricular remodeling (LV + RV) in 27% of patients, the LV in 49%, the RV in 52%, and the LA in 21% of the overall population. A second relevant finding was that HDFs offered complementary mechanistic information regarding intraventricular flow coordination and myocardial–blood coupling during cardiac reverse remodeling.

The third finding is that remodeling occurred without significant sex-related differences. In addition, a concurrent improvement of functional NYHA Class and NT-proBNP was observed, supporting a coherent link between cardiac reverse remodeling and clinical impact. Overall, the consistent and concordant changes observed across multiple echocardiographic and clinical markers support the presence of a relevant treatment-associated reverse remodeling signal, even if causal inference is limited by observational and real-world design. To enhance interpretability and reduce clinical heterogeneity, the analysis was restricted to patients with chronic HF and baseline LV-EF ≤ 45%, a population in whom reverse remodeling is more likely to occur and can be more accurately quantified over follow-up. Furthermore, the use of stringent composite criteria likely improved the specificity of reverse remodeling classification, although it may have underestimated the overall prevalence of remodeling by excluding modest but potentially relevant changes.

Although the observed effects may have been influenced by concomitant guideline-directed HF therapies and by the baseline severity of systolic dysfunction, these data reflect a real-world treatment setting in which SGLT2i are added on top of stable background therapy.

For clarity, we will discuss the findings chamber by chamber.

### 4.1. Left Ventricle Remodeling

Baseline echocardiographic assessment showed a generally reduced LV-EF with moderately dilated LV volumes, consistent with a predominant HFrEF population.

Following initiation of SGLT2i therapy, significant improvements were observed across most LV parameters within six months. Although evidence from echocardiographic studies is not entirely consistent, a beneficial effect of SGLT2i on LV remodeling has been widely reported and our findings are in line with previous literature showing improvements in both systolic and diastolic function [11]. In the present study, LV-EDV, LV-ESV and the E/e’ decreased, while LV-EF, LV-GLS, and LV-GCS increased. Prior studies have shown heterogeneous results. in Omar et al.’s study, Empaglifozin demonstrated efficacy in reducing LV-EDV and LV-ESV with no significant association with LV-EF and LV-GLS improvement [15], while Zhang et al.’s metanalysis showed a significant reduction in LV-EDV, LV-ESV and a significant improvement of LV-EF without significant differences in LV-GLS after SGLT2i therapy [16].

Pastore et al.’s study demonstrated a significant improvement of diastolic function with a significant decrease in E/e’ and a significant improvement of LV-GLS [17].

Compared with previous studies, we adopted a stringent composite definition to identify LVRR, requiring the concurrent improvement of three parameters—two reflecting systolic function (LVEF and LV-GLS) and one reflecting LV volume (LV-ESV). Despite the strictness of this definition, LVRR was observed in 33 patients (49%), highlighting a meaningful remodeling signal in this real-world cohort. 

Furthermore, for the first time in the context of SGLT2i therapy, we explored HDFs, which represent a novel and validated approach to characterize intraventricular force orientation and myocardial–blood interaction throughout the cardiac cycle. While evidence on the relationship between SGLT2i and HDFs is currently lacking, HDF-derived indices in our cohort changed in a direction consistent with global reverse remodeling. In particular, the reduction in the transverse-to-longitudinal force ratio (L–S/A–B, %) may reflect a shift towards a more physiological predominance of longitudinal (apex-to-base) forces, potentially indicating improved intraventricular flow coordination during systole and diastole. Importantly, HDFs did not show a significant association with the composite outcome in exploratory regression analyses, indicating that their value may be primarily mechanistic rather than predictive in the present dataset.

The shift toward more favorable longitudinal force distribution may be multifactorial and plausibly reflects both reductions in LV volumes and improvements in systolic performance. SGLT2i-related reductions in preload and afterload may lead to decreased ventricular wall stress and more efficient myocardial deformation, while concomitant improvements in contractile performance may further contribute to the observed redistribution of intraventricular force patterns. However, these mechanisms remain speculative and should be interpreted within the limits of an observational study.

Nevertheless, the near-significant associations observed for systolic and diastolic HDFs ratios in exploratory analyses may suggest a potential relationship between intraventricular flow realignment and reverse remodeling. Given the relatively limited sample size and the low number of remodeling events, these findings should be interpreted cautiously and considered hypothesis-generating. 

Overall, the concordant improvement across conventional LV remodeling markers and HDF-derived indices suggest that SGLT2i therapy may influence cardiac remodeling at multiple levels. 

### 4.2. Right Ventricle Remodeling

The relationship between SGLT2i and RV function remains relatively underexplored, and available imaging studies have reported inconsistent findings. Sarak et al. found no significant effect of Empagliflozin on RV function in a population of diabetic and ischemic patients [18], whereas Pastori et al. reported a significant improvement in RFW-St after Dapagliflozin therapy [17]. Similarly, Fusco et al. described a favorable effect of Dapagliflozin after one year in terms of RV-FAC and RFW-St [19], while Camçi et al. observed improvements in both TAPSE and RV-FAC [20]; conversely, Mustapic et al. reported a significant change only in RFW-St [21].

In our cohort, SGLT2i initiation was associated with a significant improvement in RV performance, as reflected by RV-GLS, RV-FAC and RFW-St, while TAPSE did not significantly change. This finding may be explained by the fact that baseline TAPSE was largely preserved and, as a surrogate of basal longitudinal RV motion, may be less sensitive to subtle or early changes compared with deformation indices and RV-FAC. From a pathophysiological perspective, potential mechanisms include reductions in LV filling pressures and pulmonary congestion, improved LV diastolic function and interventricular interaction, and a decrease in pulmonary vascular load, which may ultimately reduce RV afterload and improve RV function.

Compared with previous reports, we adopted a stringent composite definition of RVRR, requiring the concurrent improvement of three functional parameters (RV-FAC, RV-GLS, and RFW-St), thereby increasing specificity and limiting misclassification due to variability in single measures. Despite this conservative definition, RVRR was observed in 35 patients (52%).

### 4.3. Left Atrium Remodeling

Several studies have reported an improvement in LA remodeling after SGLT2i therapy, although results remain heterogeneous. Figal et al. described a reduction in indexed LAV after 6 months of Dapagliflozin, occurring alongside LVRR [22], and similar findings were reported in the meta-analysis by Fan et al. [23]. Conversely, Pourafkari et al. observed no significant changes in LAV and function after Empagliflozin [24], while the meta-analysis by Savage et al. confirmed inconsistent evidence regarding LA structural remodeling [25]. Only a limited number of small studies have specifically assessed LA functional parameters, generally reporting favorable changes after SGLT2i therapy [26,27].

In addition, some reports have suggested a reduction in atrial fibrillation (AF) burden following SGLT2i initiation, potentially mediated by decreases in LA pressure/volume overload and atrial fibrosis [28]; however, this association remains debated [29].

In our cohort, SGLT2i therapy was associated with a significant improvement in LA function, as reflected by LA strain parameters and LA-EF, whereas LA-ESV did not significantly change. This apparent dissociation may reflect the possibility that LA functional indices are more sensitive to early hemodynamic changes, including reductions in LV filling pressures and pulmonary congestion, and may recover earlier than structural reverse remodeling becomes detectable. Conversely, LA volumes may represent more advanced and long-standing structural remodeling and fibrosis, which may be less reversible and might require longer follow-up to show significant regression. Notably, at baseline LA dilatation appeared more prominent than the degree of functional impairment.

To increase specificity and reduce misclassification due to variability of single measures, we adopted a stringent composite endpoint for LARR, requiring concurrent improvement in two functional parameters (LA-EF and LA-GLS) and a one-dimensional parameter (LA-ESV). Despite this conservative definition, LARR was observed in 14 patients (21%). Nevertheless, given the real-world observational design, residual confounding cannot be excluded, as LA indices may be influenced by rhythm status, loading conditions, and changes in concomitant therapy.

### 4.4. Sex Differences

After sex stratification, male and female patients showed similar changes in echocardiographic parameters across all three chambers, including dimensions, systolic function, strain and HDFs (all *p* > 0.05). No significant sex-related differences in RR were observed in this cohort. Given the limited sample size, these exploratory findings should be interpreted with caution; however, they suggest that the remodeling response to SGLT2i may be broadly consistent across sexes in a real-world HF population. To the best of our knowledge, this is among the first imaging studies reporting comparable multi-chamber remodeling in males and females after SGLT2i initiation.

### 4.5. Clinical and Bio-Humoral Remodeling

We also assessed clinical response by evaluating changes in NHYA Class and NT-proBNP levels between baseline (T0) and follow-up (T1), as previously reported in SGLT2i studies [30]. Consistently, after 6 months of SGLT2i therapy, NT-proBNP significantly decreased in the overall population (*p* = 0.005). NYHA Class improved, with a reduction in the portion of patients in higher classes (NYHA Class > II), decreasing from 27% to 18%, although it did not reach statistical significance probably due to the small sample size (*p* = 0.21). Overall, the concordant improvement in clinical and biomarker parameters supports the clinical relevance of the observed echocardiographic RR.

The observed hospitalization and mortality rates appear to be lower than those reported in large HF registries. For example, data from the ESC Heart Failure Long-Term Registry and H2-registry have shown that 1-year mortality in real-world HF populations generally ranges between approximately 8% to 15%, while HF hospitalization rates are commonly reported in the range of approximately 30% to 50%, depending on disease severity, clinical setting, and phenotype [31,32]. Although a direct causal relationship cannot be established, these findings suggest that the observed 12-month clinical outcomes are at least not inferior to those reported in the existing literature.

### 4.6. Limitations and Future Perspectives

Despite the promising findings, this study has several limitations. Firstly, the lack of a control group and the single-center design limit causal inference and the generalizability of the findings, reducing our ability to conclusively attribute the observed changes in echocardiographic and clinical parameters solely to the therapy. The relatively limited sample size and the low number of remodeling events limited the possibility of performing robust internal validation analyses of the exploratory logistic regression models. Moreover, the observed improvements may also be influenced by regression to the mean, the natural course of the disease, or concomitant optimization of HF therapy. However, as the large majority of patients with HF are currently treated with SGLT2i, subjects not receiving this treatment are increasingly uncommon.

Another limitation is the relatively small sample size, particularly in the female subgroup. Larger studies with more balanced sex distributions are needed to better understand the underlying mechanisms of sex-based differences in treatment efficacy.

Furthermore, our study did not assess other outcomes such as quality of life and exercise capacity by CPET which would provide a more comprehensive insight into the benefits of therapy.

Finally, while the 6-month follow-up is valuable, longer follow-up periods are needed to assess the sustainability of the observed improvements and the long-term effects of SGLT2i in HF.

## 5. Conclusions

This study provides echocardiographic evidence of significant cardiac RR after 6 months of SGLT2i therapy in a real-world HF cohort, involving three cardiac chambers—from the left to the right side. These changes were accompanied by improved intraventricular flow coordination and more efficient myocardial–blood coupling, as captured by novel parameters such as HDFs. No significant sex-related differences were observed. Long-term and randomized studies are necessary to confirm these findings.

## Figures and Tables

**Table 1:** Baseline characteristics of the overall population.

Parameters	Patients n = 68
Age, years (mean ± SD)	70 ± 13
Male sex, n (%)	49 (72)
BMI, kg/m^2^ (mean ± SD)	26 ± 4
BSA, m^2^ (mean ± SD)	1.8 ± 0.2
Hypertension, n (%)	48 (71)
Dyslipidemia, n (%)	41 (60)
Diabetes, n (%)	22 (32)
Smoke Habit, n (%)	35 (51)
Ischemic Etiology, n (%)	34 (50)
NYHA class I, n (%)	5 (7)
NYHA class II, n (%)	45 (66)
NYHA class III, n (%)	17 (25)
NYHA class IV, n (%)	1 (2)
NT-proBNP, pg/mL (IQR)	1651 (663–1943)
ACEi medication, n (%)	12 (18)
ARB medication, n (%)	2 (3)
ARNi medication, n (%)	46 (68)
MRA medication, n (%)	46 (68)
BB medication, n (%)	59 (87)
Loop diuretic medication, n (%)	42 (62)

ACEi: angiotensin-converting enzyme inhibitor medication; ARB: angiotensin receptor blocker medication; ARNi: angiotensin receptor–neprilysin inhibitor medication; BB: beta-blocker medication; BMI: body mass index; BSA: body surface area; MRA: mineralocorticoid receptor antagonist medication; NT-proBNP: N-terminal pro-B-type natriuretic peptide; NYHA class: New York Heart Association.

**Table 2:** Comparison of the echocardiographic parameters between baseline (T0) and 6-month follow-up (T1).

Parameters	T0	T1	*p*-Value
LV-EDV, mL	136 (109−197)	120 (84–152)	<0.001
LV-ESV, mL	90 (66–138)	70 (49–91)	<0.001
LV-EF, %	32 (24–36)	40 (33–47)	<0.001
LV-GLS, %	−9 (−11; −7)	−13 (−16; −10)	<0.001
LV-GCS, %	−13 (−15; −11)	−19 (−22; −14)	<0.001
E/e’	12 (9–16)	9 (7–11)	0.001
RV-GLS, %	−11 (−16; −7)	−15 (−22; −11)	<0.001
RV-FAC, %	33 (24–39)	40 (30–48)	<0.001
RFW-St, %	−12 (−21; –7)	−16 (−23; −12)	0.001
TAPSE, mm	19 (18–22)	20 (18–22)	0.304
LA-ESV, mL	76 (57–97)	74 (59–97)	0.331
LA-EF, %	37 (25–48)	42 (33–57)	<0.001
LA-GLS, %	15 (9–20)	17 (10–27)	0.004
LA-GCS, %	17 (10–24)	21 (13–32)	<0.001
HDFs ratio-entire, %	31 (25–37)	28 (22–33)	0.02
HDFs ratio-systole, %	21 (16–26)	17 (12–21)	0.001
HDFs ratio-diastole, %	47 (38–51)	38 (24–44)	<0.001

E/e’: E to e-prime ratio; HDFs: hemodynamic forces; LA-EF: left atrial ejection fraction; LA-ESV: left atrial end-systolic volume; LA-GCS: left atrial global circumferential strain; LA-GLS: left atrial global longitudinal strain; LV-EDV: left ventricular end-diastolic volume; LV-EF: left ventricular ejection fraction; LV-ESV: left ventricular end-systolic volume; LV-GCS: left ventricular global circumferential strain; LV-GLS: left ventricular global longitudinal strain; RFW-St: right ventricular free wall strain; RV-FAC: right ventricular fractional area change; RV-GLS: right ventricular global longitudinal strain; TAPSE: tricuspid annular plane systolic excursion.

**Table 3:** Univariate Logistic Regression Analysis for bi-ventricular remodeling.

	Univariate		
Parameters	OR	95% CI	*p*-Value
Female sex	0.67	0.19–2.36	0.530
Age	1.01	0.96–1.05	0.927
BSA	1.70	0.16–17.98	0.657
Hypertension	2.58	0.65–10.14	0.176
Diabetes	2.04	0.65–6.11	0.224
Ischemic Etiology	0.74	0.25–2.18	0.583
NYHA class > II	2.26	0.71–7.20	0.169
NT-proBNP	1.01	0.99–1.01	0.108
E/è	0.98	0.88–1.11	0.786
EF-LA	1.01	0.97–1.05	0.590
ESV-LA	0.99	0.98–1.11	0.852
HDFs ratio entire	0.99	0.94–1.04	0.761
HDFs ratio systole	0.92	0.84–1.02	0.056
HDFs ratio diastole	0.94	0.89–1.01	0.058

BSA: Body Surface Area; E/è: ratio between early mitral inflow velocity and mitral annular early diastolic velocity; EF-LA: Left Atrial Ejection Fraction; ESV-LA: Left Atrial End-Systolic Volume; HDFs: Hemodynamic Forces; NT-proBNP: N-terminal pro-B-type Natriuretic Peptide.

**Table 4:** Univariate and Multivariate Logistic Regression Analysis for left-atrium remodeling.

	Univariate			Multivariate		
Parameters	OR	95% CI	*p*-Value	OR	95% CI	*p*-Value
Age	0.99	0.94–1.03	0.593	-	-	-
Female sex	1.59	0.45–5.55	0.469	-	-	-
Hypertension	0.69	0.20–2.40	0.563	-	-	-
Diabetes	0.28	0.06–1.36	0.113	-	-	-
AF	1.20	0.28–5.11	0.805	-	-	-
LV-EDV	1.01	1.00–1.02	0.043	1.00	0.96–1.03	0.902
LV-ESV	1.01	1.00–1.02	0.036	1.01	0.97–1.06	0.564
LV-EF	0.94	0.87–1.02	0.128	-	-	-
LV-GLS	1.01	0.83–1.21	0.960	-	-	-
LV-GCS	1.14	0.97–1.34	0.121	-	-	-
E/è	0.95	0.83–1.09	0.478	-	-	-
TAPSE	0.94	0.78–1.14	0.543	-	-	-
RV-GLS	0.97	0.89–1.06	0.517	-	-	-
RV FAC	1.00	0.95–1.07	0.894	-	-	-
HDFs ratio entire	0.98	0.93–1.04	0.531	-	-	-
HDFs ratio systole	1.02	0.94–1.10	0.661	-	-	-
HDFs ratio diastole	1.02	0.95–1.09	0.550	-	-	-

AF: Atrial Fibrillation; E/è: ratio between early mitral inflow velocity and mitral annular early diastolic velocity; HDFs: Hemodynamic Forces; LV-EDV: Left Ventricular End-Diastolic Volume; LV-EF: Left Ventricular Ejection Fraction; LV-ESV: Left Ventricular End-Systolic Volume; LV-GCS: Left Ventricular Global Circumferential Strain; LV-GLS: Left Ventricular Global Longitudinal Strain; RV-FAC: Right Ventricular Fractional Area Change; RV-GLS: Right Ventricular Global Longitudinal Strain; TAPSE: Tricuspid Annular Plane Systolic Excursion.

**Table 5:** Comparison of relative median delta [IQR] of echocardiographic parameters from baseline (T0) to 6-month follow-up (T1) between males and females.

Parameter	Male	Female	*p*-Value
LV-EDV (mL)	0.07 [0.01; 0.31]	0.14 [0.07; 0.36]	0.134
LV-ESV (mL)	0.18 [0.07; 0.45]	0.27 [0.08; 0.42]	0.561
LV-EF (%)	−0.25 [−0.48; −0.1]	−0.17 [−0.48; −0.06]	0.385
LV-GLS (%)	−0.46 [−0.87; −0.17]	−0.44 [−0.88; −0.05]	0.832
LV-GCS (%)	−0.38 [−0.78; −0.09]	−0.34 [−0.66; −0.12]	0.790
RV-GLS (%)	−0.38 [−1.04; −0.01]	−0.50 [−0.87; 0.08]	0.822
RV-FAC (%)	−0.28 [−0.56; −0.12]	−0.19 [−0.50; 0.13]	0.199
LA-EF (%)	−0.19 [−0.34; −0.03]	−0.33 [−0.77; −0.14]	0.229
LA-GLS (%)	−0.52 [−1.17; 0.14]	−0.11 [−0.71; 0.54]	0.157
LA-ESV (mL)	0.04 [−0.35; 0.18]	0.22 [−0.23; 0.44]	0.096
HDFs ratio entire (%)	0.09 [−0.06; 0.34]	0.09 [−0.36; 0.21]	0.485

HDF: Hemodynamic Forces; LV-EDV: Left Ventricular End-Diastolic Volume; LV-ESV: Left Ventricular End-Systolic Volume; LV-EF: Left Ventricular Ejection Fraction; LV-GLS: Left Ventricular Global Longitudinal Strain; LV-GCS: Left Ventricular Global Circumferential Strain; LA-EF: Left Atrial Ejection Fraction; RV-GLS: Right Ventricular Global Longitudinal Strain; RV-FAC: Right Ventricular Fractional Area Change.

## Data Availability

The original contributions presented in this study are included in the article/Supplementary Material. Further inquiries can be directed to the corresponding author.

## Supplementary Materials

The following supporting information can be downloaded at: https://www.mdpi.com/article/10.3390/jcdd13060260/s1, Table S1: Measurement variability and literature support for echocardiographic parameters used to define ventricular and atrial reverse remodeling. Table S2: Intra-operator and inter-operators’ reproducibility analysis.

## References

[1] Aimo A., Fabiani I., Vergaro G., Arzilli C., Chubuchny V., Pasanisi E.M., Petersen C., Poggianti E., Taddei C., Pugliese N.R. (2021). Prognostic value of reverse remodelling criteria in heart failure with reduced or mid-range ejection fraction. ESC Heart Fail..

[2] Prosperi S., D’Amato A., Severino P., Myftari V., Monosilio S., Marchiori L., Zagordi L.M., Filomena D., Di Pietro G., Birtolo L.I. (2023). Sizing SGLT2 Inhibitors Up: From a Molecular to a Morpho-Functional Point of View. Int. J. Mol. Sci..

[3] Vaduganathan M., Docherty K.F., Claggett B.L., Jhund P.S., de Boer R.A., Hernandez A.F., Inzucchi S.E., Kosiborod M.N., Lam C.S.P., Martinez F. (2022). SGLT-2 inhibitors in patients with heart failure: A comprehensive meta-analysis of five randomised controlled trials. Lancet.

[4] Danielson C., Lileikyte G., Ouwerkerk W., Lam C.S., Erlinge D., Teng T.K. (2022). Sex differences in efficacy of pharmacological therapies in heart failure with reduced ejection fraction: A meta-analysis. ESC Heart Fail..

[5] McDonagh T.A., Metra M., Adamo M., Gardner R.S., Baumbach A., Böhm M., Burri H., Butler J., Čelutkienė J., Chioncel O. (2021). 2021 ESC Guidelines for the diagnosis and treatment of acute and chronic heart failure. Eur. Heart J..

[6] McDonagh T.A., Metra M., Adamo M., Gardner R.S., Baumbach A., Böhm M., Burri H., Butler J., Čelutkienė J., Chioncel O. (2023). 2023 Focused Update of the 2021 ESC Guidelines for the diagnosis and treatment of acute and chronic heart failure. Eur. Heart J..

[7] Galderisi M., Cosyns B., Edvardsen T., Cardim N., Delgado V., Di Salvo G., Donal E., Sade L.E., Ernande L., Garbi M. (2017). Standardization of adult transthoracic echocardiography reporting in agreement with recent chamber quantification, diastolic function, and heart valve disease recommendations: An expert consensus document of the European Association of Cardiovascular Imaging. Eur. Heart J. Cardiovasc. Imaging.

[8] Pedrizzetti G., Arvidsson P.M., Töger J., Borgquist R., Domenichini F., Arheden H., Heiberg E. (2017). On estimating intraventricular hemodynamic forces from endocardial dynamics: A comparative study with 4D flow MRI. J. Biomech..

[9] Monosilio S., Filomena D., Luongo F., Sannino M., Cimino S., Neccia M., Mariani M.V., Birtolo L.I., Benedetti G., Tonti G. (2022). Cardiac and Vascular Remodeling After 6 Months of Therapy with Sacubitril/Valsartan: Mechanistic Insights from Advanced Echocardiographic Analysis. Front. Cardiovasc. Med..

[10] Filomena D., Cimino S., Monosilio S., Galea N., Mancuso G., Francone M., Tonti G., Pedrizzetti G., Maestrini V., Fedele F. (2022). Impact of intraventricular haemodynamic forces misalignment on left ventricular remodelling after myocardial infarction. ESC Heart Fail..

[11] Mustapic I., Bakovic D., Susilovic-Grabovac Z., Borovac J.A. (2023). Left Ventricular Systolic Function After 3 Months of SGLT2 Inhibitor Therapy in Heart Failure Patients with Reduced Ejection Fraction. J. Cardiovasc. Transl. Res..

[12] Hardegree E.L., Sachdev A., Villarraga H.R., Frantz R.P., McGoon M.D., Kushwaha S.S., Hsiao J.F., McCully R.B., Oh J.K., Pellikka P.A. (2013). Role of serial quantitative assessment of right ventricular function by strain in pulmonary arterial hypertension. Am. J. Cardiol..

[13] Tops L.F., Bax J.J., Zeppenfeld K., Jongbloed M.R., van der Wall E.E., Schalij M.J. (2006). Effect of radiofrequency catheter ablation for atrial fibrillation on left atrial cavity size. Am. J. Cardiol..

[14] Stassen J., Galloo X., Chimed S., Hirasawa K., Marsan N.A., Delgado V., van der Bijl P., Bax J.J. (2022). Clinical implications of left atrial reverse remodelling after cardiac resynchronization therapy. Eur. Heart J. Cardiovasc. Imaging.

[15] Omar M., Jensen J., Ali M., Frederiksen P.H., Kistorp C., Videbæk L., Poulsen M.K., Tuxen C.D., Möller S., Gustafsson F. (2021). Associations of Empagliflozin with Left Ventricular Volumes, Mass, and Function in Patients with Heart Failure and Reduced Ejection Fraction: A Substudy of the Empire HF Randomized Clinical Trial. JAMA Cardiol..

[16] Zhang N., Wang Y., Tse G., Korantzopoulos P., Letsas K.P., Zhang Q., Li G., Lip G.Y.H., Liu T. (2022). Effect of sodium-glucose cotransporter-2 inhibitors on cardiac remodelling: A systematic review and meta-analysis. Eur. J. Prev. Cardiol..

[17] Pastore M.C., Stefanini A., Mandoli G.E., Piu P., Diviggiano E.E., Iuliano M.A., Carli L., Marchese A., Martini L., Pecere A. (2024). Dapagliflozin Effects on Cardiac Deformation in Heart Failure and Secondary Clinical Outcome. JACC Cardiovasc. Imaging.

[18] Sarak B., Verma S., David Mazer C., Teoh H., Quan A., Gilbert R.E., Goodman S.G., Bami K., Coelho-Filho O.R., Ahooja V. (2021). Impact of empagliflozin on right ventricular parameters and function among patients with type 2 diabetes. Cardiovasc. Diabetol..

[19] Fusco F., Scognamiglio G., Abbate M., Merola A., Grimaldi N., Ciriello G.D., Sarubbi B. (2024). Dapagliflozin in patients with a failing systemic right ventricle: Results from the DAPA-SERVE trial. JACC Heart Fail..

[20] Çamcı S., Yılmaz E. (2022). Effects of sodiumglucose co-transporter-2 inhibition on pulmonary arterial stiffness and right ventricular function in heart failure with reduced ejection fraction. Medicina.

[21] Mustapic I., Bakovic D., Susilovic Grabovac Z., Borovac J.A. (2022). Impact of SGLT2 inhibitor therapy on right ventricular function in patients with heart failure and reduced ejection fraction. J. Clin. Med..

[22] Pascual-Figal D.A., Zamorano J.L., Domingo M., Morillas H., Nuñez J., Cobo Marcos M., Riquelme-Pérez A., Teis A., Santas E., Caro-Martinez C. (2023). Impact of dapagliflozin on cardiac remodelling in patients with chronic heart failure: The DAPA-MODA study. Eur. J. Heart Fail..

[23] Fan G., Guo D.L. (2023). The effect of sodium-glucose cotransporter-2 inhibitors on cardiac structure remodeling and function: A meta-analysis of randomized controlled trials. Eur. J. Intern. Med..

[24] Pourafkari M., Connelly K.A., Verma S., Mazer C.D., Teoh H., Quan A., Goodman S.G., Rai A., Ng M.Y., Deva D.P. (2024). Empagliflozin and left atrial function in patients with type 2 diabetes mellitus and coronary artery disease: Insight from the EMPA-HEART CardioLink-6 randomized clinical trial. Cardiovasc. Diabetol..

[25] Savage P., Watson C., Coburn J., Cox B., Shahmohammadi M., Grieve D., Dixon L. (2024). Impact of SGLT2 inhibition on markers of reverse cardiac remodelling in heart failure: Systematic review and meta-analysis. ESC Heart Fail..

[26] El-Saied S.B., El-Sherbeny W.S., El-Sharkawy S.I. (2023). Impact of sodium glucose co-transporter-2 inhibitors on left atrial functions in patients with type-2 diabetes and heart failure with mildly reduced ejection fraction. Int. J. Cardiol. Heart Vasc..

[27] Sehly A., He A., Ihdayhid A.R., Grey C., O’Connor S., Green G., Erickson M., Rankin J.M., Fegan P.G., Yeap B.B. (2024). Early SGLT2 inhibitor use is associated with improved left atrial strain following acute coronary syndrome. Acta Cardiol..

[28] Butt J.H., Kondo T., Jhund P.S., Comin-Colet J., de Boer R.A., Desai A.S., Hernandez A.F., Inzucchi S.E., Janssens S.P., Kosiborod M.N. (2022). Atrial Fibrillation and Dapagliflozin Efficacy in Patients with Preserved or Mildly Reduced Ejection Fraction. J. Am. Coll. Cardiol..

[29] Zhang H.D., Ding L., Mi L.J., Zhang A.K., Zhang K., Jiang Z.H., Yu F.Y., Yan X.X., Shen Y.J., Tang M. (2024). Sodium-glucose co-transporter-2 inhibitors for the prevention of atrial fibrillation: A systemic review and meta-analysis. Eur. J. Prev. Cardiol..

[30] McMurray J.J.V., Solomon S.D., Inzucchi S.E., Køber L., Kosiborod M.N., Martinez F.A., Ponikowski P., Sabatine M.S., Anand I.S., Bělohlávek J. (2019). Dapagliflozin in Patients with Heart Failure and Reduced Ejection Fraction. N. Engl. J. Med..

[31] Chioncel O., Lainscak M., Seferovic P.M., Anker S.D., Crespo-Leiro M.G., Harjola V.P., Parissis J., Laroche C., Piepoli M.F., Fonseca C. (2017). Epidemiology and one-year outcomes in patients with chronic heart failure and preserved, mid-range and reduced ejection fraction: An analysis of the ESC Heart Failure Long-Term Registry. Eur. J. Heart Fail..

[32] Leiner J., König S., Nitsche A., Hohenstein S., Nagel J., Seyfarth M., Baberg H., Lauten A., Neuser H., Staudt A. (2025). A multicentre registry of hospitalized patients with acute and chronic heart failure: Study design of the H2-registry. ESC Heart Fail..

