# Supplementary material for: Multi-Chamber Reverse Remodeling and Hemodynamic Force Realignment After SGLT2 Inhibitor Initiation in Real-World Heart Failure
Source: J Cardiovasc Dev Dis. 2026 Jun 11;13(6):260. doi: 10.3390/jcdd13060260 (PMC13300219; doi:10.3390/jcdd13060260)
Supplement: Supplementary file 1 [file jcdd-13-00260-s001.zip › jcdd-4287359-supplementary.pdf]

SUPPLEMENTARY DATA

Multi-Chamber Reverse Remodeling and Hemodynamic Force Realignment After SGLT2 Inhibitor Initiation in Real-World Heart Failure

The following data has been provided as supporting material due to limited space in the main manuscript:

Supplementary Table S1. Measurement variability and literature support for echocardiographic parameters used to define ventricular and atrial reverse remodeling.

Chamber	Parameter	Measurement variability	Literature-supported RR threshold	Ref.
**LVRR**	LV-ESV	~10%	≥15% reduction	9
	LV-EF	~5–10%	≥10% absolute improvement	9
	LV-GLS	~2–3%	≥3% absolute improvement	11
**RVRR**	RVW-st	~2.5–4.5%	≥5% absolute increase	12
**LARR**	LAVi	~5-8%	≥15% reduction	13
	LA-GLS	~3-5%	≥5% absolute increase	14

LA-GLS: left atrial global longitudinal strain; LARR: left atrial reverse remodeling; LAVi: left atrial volume index; LV-EF: left ventricular ejection fraction; LV-ESV: left ventricular end-systolic volume; LV-GLS: left ventricular global longitudinal strain; LVRR: left ventricular reverse remodeling; RVW-st: right ventricular free-wall longitudinal strain; RR: reverse remodeling; RVRR: right ventricular reverse remodeling

Bibliography for Supplementary Table 1:

9 - Monosilio, S.; Filomena, D.; Luongo, F.; Sannino, M.; Cimino, S.; Neccia, M.; Mariani, M.V.; Birtolo, L.I.; Benedetti, G.; Tonti, G.; et al. Cardiac and Vascular Remodeling After 6 Months of Therapy With Sacubitril/Valsartan: Mechanistic Insights From Advanced Echocardiographic Analysis. Front. Cardiovasc. Med. 2022, 9, 883769. https://doi.org/10.3389/fcvm.2022.883769. PMID: 35665260; PMCID: PMC9157573.

11- Mustapic, I.; Bakovic, D.; Susilovic-Grabovac, Z.; Borovac, J.A. Left Ventricular Systolic Function After 3 Months of SGLT2 Inhibitor Therapy in Heart Failure Patients with Reduced Ejection Fraction. *J. Cardiovasc. Transl. Res.* **2023**, *16*, 987–998. https://doi.org/10.1007/s12265-023-10389-3. PMID: 37155137; PMCID: PMC10166024.

12- Hardegree, E.L.; Sachdev, A.; Villarraga, H.R.; Frantz, R.P.; McGoon, M.D.; Kushwaha, S.S.; Hsiao, J.F.; McCully, R.B.; Oh, J.K.; Pellikka, P.A.; et al. Role of serial quantitative assessment of right ventricular function by strain in pulmonary arterial hypertension. *Am. J. Cardiol.* **2013**, *111*, 143–148. https://doi.org/10.1016/j.amjcard.2012.08.061. PMID: 23102474.

13- Tops, L.F.; Bax, J.J.; Zeppenfeld, K.; Jongbloed, M.R.; van der Wall, E.E.; Schalij, M.J. Effect of radiofrequency catheter ablation for atrial fibrillation on left atrial cavity size. *Am. J. Cardiol.* **2006**, *97*, 1220–1222. https://doi.org/10.1016/j.amjcard.2005.11.043. PMID: 16616029.

14- Stassen, J.; Galloo, X.; Chimed, S.; Hirasawa, K.; Marsan, N.A.; Delgado, V.; van der Bijl, P.; Bax, J.J. Clinical implications of left atrial reverse remodelling after cardiac resynchronization therapy. *Eur. Heart J. Cardiovasc. Imaging* **2022**, *23*, 730–740. https://doi.org/10.1093/ehjci/jeac042. PMID: 35213709; PMCID: PMC9291382.

Supplementary Table S2. Intra-operator and inter-operators' reproducibility analysis

Parameters	Intra-observer Agreement*	P	Inter-observer Agreement*	P
**LV-GLS, %**	0.985 (CI 0.960-0.995)	<0.001	0.934 (CI 0.822-0.976)	<0.001
**RV-GLS, %**	0.981 (CI 0.947-0.993)	<0.001	0.934 (CI 0.821-0.976)	<0.001
**LA-GLS, %**	0.980 (CI 0.943-0.993)	<0.001	0.955 (CI 0.880-0.984)	<0.001
**HDFs entire AB, %**	0.987 (CI 0.963-0.995)	<0.001	0.988 (CI 0.968-0.996)	<0.001
**HDFs entire LS, %**	0.978 (CI 0.939-0.992)	<0.001	0.969 (CI 0.917-0.989)	<0.001
**HDFs entire ratio, %**	0.973 (CI 0.925-0.990)	<0.001	0.954 (CI 0.877-0.984)	<0.001

*Intralass coefficient (ICC) with confidence intervals and p-values are reported

LA-GLS: Left Atrial Global Longitudinal Strain; **LV-GLS**: Left Ventricular Global Longitudinal Strain; RV-**GLS**: Right Ventricular Global Longitudinal Strain; **HDFs entire AB:** Haemodynamic Forces on the entire cardiac cycle with direction Apex to Base; **HDFs entire LS:** Haemodynamic Forces on the entire cardiac cycle with direction Lateral to Septal; **HDFs entire ratio:** Haemodynamic Forces LS/AB ratio.
